# Data models, representation and adequacy-for-purpose

**DOI:** 10.1007/s13194-020-00345-2

**Published:** 2021-01-29

**Authors:** Alisa Bokulich, Wendy Parker

**Affiliations:** 1 Department of Philosophy, Boston University, 745 Commonwealth Avenue, Room 516, Boston, MA 02215 USA; 2 Department of Philosophy, Virginia Tech, 229 Major Williams Hall (0126), Blacksburg, VA 24060 USA

**Keywords:** Data, Data models, Data processing, Scientific models, Fit-for-purpose, Legacy data, Climate science, Astrophysics, Geosciences, Pragmatism

## Abstract

We critically engage two traditional views of scientific data and outline a novel philosophical view that we call the *pragmatic-representational (PR) view of data*. On the PR view, data are representations that are the product of a process of inquiry, and they should be evaluated in terms of their adequacy or fitness for particular purposes. Some important implications of the PR view for data assessment, related to misrepresentation, context-sensitivity, and complementary use, are highlighted. The PR view provides insight into the common but little-discussed practices of iteratively reusing and repurposing data, which result in many datasets’ having a phylogeny—an origin and complex evolutionary history—that is relevant to their evaluation and future use. We relate these insights to the open-data and data-rescue movements, and highlight several future avenues of research that build on the PR view of data.

## Introduction

Philosophers of science now commonly understand theoretical models to be *representations* of real or imagined targets, with a variety of nuanced perspectives on how best to understand the nature of this representational relationship (e.g., Frigg and Nguyen 2017; Frigg and Hartmann 2020). In conjunction with the rise of this representational perspective, there has also been a *pragmatic* turn: many philosophers now emphasize that scientific modeling is an activity undertaken by agents with specific goals and purposes in mind, such as the prediction or explanation of a particular phenomenon. That is, models are not just representations; they are also tools, which are constructed or selected, and manipulated, with an eye toward achieving specific epistemic or practical purposes (e.g., Morrison and Morgan 1999; Giere 2004, 2010; Boon and Knuuttila 2009; Parker 2010; Knuuttila 2011; Currie 2018; Boon 2020). A closely-related view of model evaluation proposes that models be judged in terms of their adequacy or fitness for particular purposes, rather than by comparison to some abstract standard of perfect representation (Parker 2010, 2020a; Currie 2018; see also Teller 2001; NRC 2007; Taper et al. 2008).

This pragmatic, representational perspective on scientific models has, so far, been articulated and adopted almost exclusively in the context of *theoretical* modeling, where model construction begins from theoretical principles or other general assumptions about the workings of a system or phenomenon. Familiar examples include Newtonian models of the pendulum, fluid dynamical models of earth’s atmosphere, and predator-prey models in ecology. When it comes to another important class of models used in science—*data models*—a pragmatic representational view has not yet been similarly developed or defended.^1^ Data models are often described as cleaned-up, smoothed or otherwise-processed versions of data (Suppes 1962; Frigg and Hartmann 2020). Examples of data models include global climate datasets synthesized from a huge number of local temperature records, filtered and colored astronomical images of galaxies, and graphs showing support for a political candidate over time obtained by averaging the results from several polls.^2^ While data models are sometimes characterized as representations (e.g., Harris 2003; Leonelli 2016), the idea that they should be evaluated in terms of their adequacy-for-purpose is rarely expressed; the implicit measure of quality, both in scientific and philosophical discussions, usually remains that of closeness to a perfect mirroring of the world.

We argue that a pragmatic, representational view should also be adopted in the context of data modeling and, indeed, for data themselves. Our aims in what follows are to motivate and outline such a view and to discuss some salient features of data practices from its perspective. In Section 2, we critically examine two common ways of thinking about data and data modeling—the mirroring view and the set theoretic view—and motivate the need for an alternative understanding of data. In Section 3, we present our pragmatic-representational (PR) view: data and data models are representations that should be evaluated in terms of their adequacy for particular purposes. We illustrate this view with an example from climate science. In Section 4, we draw out some significant implications for the practice of data assessment, related to misrepresentation, context-sensitivity and complementary use, which parallel insights that have emerged from a pragmatic, representational perspective on theoretical modelling. In Section 5, we discuss the dynamic evolution of data models from the perspective of the PR view. In particular, we call attention to the iterative reuse and repurposing of data, illustrating with examples from astrophysics. Such practices reveal that data models often have a complex evolutionary history, which can be highly relevant to their evaluation. We draw together these insights of the PR view of data and conclude in Section 6.

## Challenging two unhelpful views about data

In developing a philosophical view of data that is accountable to scientific practice, there are two extreme positions that we argue are unhelpful. The first is that data are an unmediated window onto the world, whose epistemic reliability is given. This view fails to adequately distinguish data from the world, in effect identifying data with reality and leaving little room for the recognition that data can misinform. At the other extreme is the philosophical view that data are abstract set-theoretic structures that can only be related to other abstract set-theoretic structures (e.g., via isomorphisms). This view also fails to adequately account for the relationship between data and the world, but this time it is the world that is lost. In this section, we flesh out key problems with each of these views in turn.

On the first view, data just are pieces of reality, or, marginally better, they provide direct access to reality by reflecting or mirroring it. This view treats data as both given and always epistemically privileged. While the mirror view guides much of our everyday thinking about data, it has long been challenged by philosophers. Thomas Kuhn, for example, famously notes that data are not “the given of experience” but rather “the collected with difficulty” (Kuhn 1996 [1962], p. 126). More recently, this insight has been further developed and defended by Sabina Leonelli, who argues that “despite their scientific value as ‘given,’ data are clearly made. They are the results of complex processes of interaction between researchers and the world” (2016, p. 71; see also Humphreys 2013). The mirror view of data has also been challenged by some scientists, such as the theoretical biologist Robert Rosen:

[A]ny measurement, however comprehensive, is an act of *abstraction*, an act of replacing the thing measured (e.g., the natural system . . .) by a limited set of numbers. Indeed, there can be no greater act of abstraction than the collapsing of a phenomenon in [nature] down to a single number, the result of a single measurement. From this standpoint, it is ironic indeed that a mere observer regards oneself as being in direct contact with reality and that it is ‘theoretical science’ alone that deals with abstractions. (Rosen 1991, p. 60)

As Rosen rightly points out, the widespread failure to appreciate these abstractions involved in the collection and production of data has perpetuated an uncritical view of data.^3^

These insights—that data are constructed through our complex interactions with the world, often involving significant abstraction—undermine the naive intuition that we can simply identify data with the world. This is not to say that data have no substantial connection to, or anchoring in, reality, but the extent to which any given datum can inform us about the world is something that should be assessed and not assumed. While this latter point seems uncontroversial, its full implications for a philosophy of data remain to be fully explored.

At the other extreme, much of the philosophical work that has been done on data has either explicitly or implicitly assumed a view of data that is arguably too disconnected from the world. One of the most influential early papers in the philosophy of data is Patrick Suppes’ (1962) “Models of Data.” In this paper, Suppes introduces the seminal notion of a “data model” and the related concept of a hierarchy of models bridging data and theory. He notes that, rather than the “raw” data, what scientists are primarily interested in is a *model of the data*—a processed and abstracted version of the data that has been subjected to appropriate statistical and other analysis.

While Suppes is right to call attention to the central importance of data models, what has often been overlooked or unchallenged in subsequent discussions is that Suppes’ view of data models is tied specifically to the semantic conception of theories and the corresponding “instantial” view of models.^4^ The notion of model that Suppes adopts when characterizing both theoretical and data models is the logician’s notion of a model as a set-theoretic structure. Following Alfred Tarski, he defines a model of a theory, T, as a possible realization of T such that all valid sentences of T are satisfied. A theory, on this semantic conception, just is a family of set-theoretic models. In an earlier article, Suppes writes, “I claim that the concept of model in the sense of Tarski may be used without distortion and as a fundamental concept in all of the disciplines . . . In this sense I would assert that the meaning of the concept of model is the same in mathematics and the empirical sciences” (Suppes 1960, p. 289).

Subsequent studies of modeling practice, however, have not born this view out. Instead, philosophers today typically characterize theoretical models as *representations* of concrete physical entities, and of course models may even be physical entities themselves (as in the case of scale ‘table top’ models) (see, e.g., Bokulich and Oreskes 2017; Frigg and Nguyen 2017; Frigg and Hartmann 2020). This representational turn in philosophical understanding of scientific models was to a significant extent spurred by the work of Ron Giere (1999, 2004, 2010). In his critique of Suppes’ (1960) paper, he highlights several issues in scientific modeling that the logician’s “instantial” view of models is ill-equipped to handle, and argues instead for a representational view: “I call my understanding of models *representational* because it takes models not primarily as providing a means for interpreting formal systems, but as tools for representing the world” (Giere 1999, p. 44).

Giere’s critique, however, centers on the instantial view of *theoretical* models. Yet Suppes adopts the same instantial view when he speaks of “models of data.” Suppes writes, “Models of the data . . . are then defined . . . in terms of possible realizations of data. As should be apparent, from a logical standpoint possible realizations of data are defined in just the same way as possible realizations of the theory” (Suppes 1962, p. 253). Surprisingly, this aspect of Suppes’ view has gone unchallenged (or perhaps not fully recognized) in many subsequent discussions of his views on data models. Even Giere, in the same (1999) paper that challenges Suppes’ instantial account of theoretical models, has a section on “Models and Data” where he endorses Suppes’ notions of models of data, and related hierarchy, without extending his critique. The same elision happens in Sabina Leonelli’s (2016) discussion of Suppes’ models of data in her book on *Data-Centric Biology*.^5^

The Suppesian construal of data models as set-theoretic (or other abstract mathematical) structures, however, is not innocuous. It leaves the relation between data models and the world at best unanalyzed, and at worst erased. As Katherine Brading and Elaine Landry have argued:

The term 'model' in science is, of course, replete with connotations of representation, and the temptation in the past has perhaps been for the semantic view of theories, with its use of Tarskian models (which, to repeat, are truth makers and *not* representations), to piggyback on this required representational role. In our view this is not acceptable (Brading and Landry 2006, p. 577).

Drawing on the distinction between presentation and representation, they contend that the relation between data models and the world cannot be captured solely in terms of the presentation of shared structure. The concern is that the Suppesian notion of data model, in resting on the notion of models as truth makers (not representations) gives no account of how data models are *about* the world.^6^

Indeed, a consequence of Suppes’ approach to data models, as Roman Frigg and James Nguyen point out, is that “[theoretical] models don’t represent planets, atoms, or populations; they represent data that are gathered when performing measurements on planets, atoms, or populations” (Frigg and Nguyen 2017, pp. 71–72). Yet we want our scientific theories and models to tell us about the world, not just about the abstract, formal structure of our data; no matter how many rungs we have in our data model hierarchy, at some point we need our ladder to reach the ground.

This problem becomes particularly acute in some recent incarnations of Suppes’ view, such as Bas van Fraassen’s scientific structuralism, where the relation between data models and the world is not just unaccounted for, but in effect erased. The easiest way to see this is through van Fraassen’s own example of a deer population, which he presents as follows. Suppose that I have represented the growth of the deer population in Princeton with a graph, and that theory T provides models that fit well with the structure displayed in the graph. Someone might object, however, that what we are really interested in is the fit of the theory with the actual deer population in the world, not just with the graph of our data. van Fraassen here responds:

[*T*]*here is no leeway for me in this context*, short of withdrawing my graph altogether. Since this is *my* representation of the deer population growth, there is *for me* no difference between the question of whether T fits the graph and the question whether T fits the deer population growth. (van Fraassen 2008, p. 256; emphasis original)

In other words, for the scientist who accepts a data model as *her* data model, there is a kind of identification of the data model (e.g., the structure indicated by the deer population graph) and the world (the structure of the actual population of deer in Princeton), such that the distinction between them is collapsed. The question of how—or how well—the data model represents the world can no longer be broached.

van Fraassen is quite aware that one might object to this move and appropriately labels it the Loss of Reality Objection (2008, p. 258). He tries to dissolve the objection by arguing that there is a “pragmatic tautology” between a theoretical model adequately representing the world and it adequately representing the data model—a move which has been criticized by James Nguyen (2016). We too reject this move; as we illustrate in later sections, scientists are not only able to—but in fact routinely do—draw a meaningful distinction between their data models and the world. Indeed, the iterative process of trying to find ways to correct the data and better bridge this gap is a central component of scientific practice.

Our aim here is not to offer a comprehensive analysis or critique of Suppes’ and van Fraassen’s accounts, but simply to point out that the common, superficial endorsement of their views on data models has been far too sanguine. Like the mirror view of data, an abstract structuralist set-theoretic view of data is limited in its ability to make sense of scientific practice. What is needed instead is a view of data that leaves room for both the researcher and the world—as well as the complex iterative interplay between them—in constructing data about the world. We now attempt to offer such a view.

## The pragmatic-representational (PR) view of data

In this section we introduce the key elements of our PR view of data, beginning first with a discussion of data and data models as representations that are products of a process of inquiry (Section 3.1). Next we turn to the issue of data quality, advocating an adequacy-for-purpose approach to data evaluation (Section 3.2). Finally, we illustrate these elements of our PR view of data with an example from climate science (Section 3.3).

### Data as representations

We understand *data* to be records of the results of a process of inquiry that involves interacting with the world. These records can take various forms—computer files storing numerical values, inscriptions on paper, photographs, etc.^7^ Researchers collect, select and use data that they have reason to believe can, perhaps with further processing and manipulation, be informative about aspects of the world that interest them. Usually, the expectation that data can be informative in this way is grounded in the belief that the aspects of the world that are of interest have played some causal role in generating the results that the data are meant to document (see also Woodward 2010, p. 793; Leonelli 2019). This does not, of course, rule out that other factors, such as features of the instruments, observers, and environmental conditions, have also played a role in the production of the data. Indeed, recognizing the influence of these additional factors is often crucial to the evaluation and effective use of data, as we will show repeatedly in what follows.

Data are representational in at least the minimal sense that they are *taken to be about* one or more aspects of the world, namely, those thought to be involved in a particular process of inquiry. In most cases, however, conventions of interpretation, metadata, or simply familiarity with the process by which the data were produced, will lead a researcher to attribute more *specific representational content* to the data. For example, the numerical value “35” inscribed in a weather station’s log book is understood by a meteorologist to represent the depth of water (in millimeters) contained in a particular rain gauge at a given time, as recorded by a particular observer. It might also be understood to represent the depth of rainfall that fell in that location over a certain period, since that is what rain gauges are designed to measure.

Such representational content will have a certain level of *accuracy*: it will be closer to or farther from the “true value”, however this might be understood (see Tal 2011; Teller 2018). Following the standard analogy, if we think of the true value as the bullseye of a dartboard, then accuracy is how close to the bullseye the dart (measured value) lands. It will also have a certain *resolution*: data reporting rain gauge collections to the nearest millimeter have finer resolution than data reporting only to the nearest centimeter. Continuing the analogy, resolution refers to how thin or thick the point of the dart is. We can also speak of the *precision* of the process of inquiry that generates the data: how closely the results of repeated applications of that process would cluster together.^8^ One can have high precision with low accuracy, such as when a number of darts land closely together in a small region of the dartboard that is far from the bullseye. Depending on the question that researchers seek to answer with the data, a certain minimum level of precision, accuracy, or resolution might be required.

Data are representational, but this does not mean that they can only be informative about one aspect of the world, such as that intended by the original data collector or that which they represent if taken at face value according to conventions of interpretation. For example, if the amount of rain collected by a rain gauge is influenced by both the actual rainfall and the ambient windspeed, then a datum reporting that collected amount has the potential to be informative about either of these aspects of the world. One meteorologist might use a wind-loss equation, in conjunction with data on windspeed, to correct the rain gauge reading for wind effects and arrive at a more accurate estimate of rainfall; another meteorologist who already has a highly accurate estimate of that rainfall might use the rain gauge reading, in conjunction with the same wind-loss equation, to estimate the average wind speed during the rainfall. An interfering factor for the first meteorologist (i.e., wind) is the target of inquiry for the second.

As this example suggests, and as Leonelli (2019, p. 17) has emphasized, data do not have fixed evidential value. What data are taken to provide evidence about can change from context to context, depending on the interests, background knowledge, and other resources available to researchers. Indeed, the evidential capacity of data can often be extended far beyond what was envisioned by the initial data collector (see also Section 5.2 below), as scientific knowledge develops over time, as researchers learn about additional factors that influenced the data, or as the data can be related to additional quantities of interest in a systematic way. Nonetheless, the evidential value of data is still *constrained* by the fact that they are the product of a particular set of causal factors and not others. We cannot reasonably take rain gauge data to provide evidence about the mass of a distant asteroid, for example.

Researchers sometimes distinguish between “raw data” on the one hand and “data models” or “data products” on the other. While “raw data” is often taken to mean “unprocessed” outputs of instruments or observing procedures, this way of thinking is increasingly challenged as instruments themselves embed more and more computational processing, from averaging to theory-based calculations (see e.g. Humphreys 2013, 2014). Moreover, in practice, such terminology frequently tracks not an absolute or intrinsic difference, but a relative one: datasets that are taken as input to a given study might be considered “raw” data, even if they are the product of substantial prior processing; when the data then undergo further processing (e.g., synthesizing, filtering, correcting, or smoothing) in order to make them better serve the study’s aims, the researchers consider the result to be a *data model* or *data product*. In the present discussion, we will generally avoid talk of “raw” data. We will understand “data models” to refer to datasets or other entities—graphs, charts, equations, etc.—that are produced by processing other data or data models. Following practitioners, we will sometimes use the label *data product* for a data model whose production has involved substantive processing, such as a transformation from one physical quantity to another, or the filling in of gaps using theoretical calculation, or the synthesis of data with simulation output.

Like data themselves, data models are representations.^9^ Suppose the rainfall datum mentioned above is corrected for loss due to wind effects, with the aim of arriving at a more accurate estimate of rainfall depth. The wind-corrected estimate—a representation of rainfall depth, say 41 mm—might then be considered a data model, as could a dataset consisting of a time series of such corrected estimates, or even a graph obtained by plotting those estimates and fitting a curve (e.g., a line) to them; the latter might be taken to represent the contribution of a particular causal factor to changes in rainfall in the locale during the period. As the latter example suggests, data models often are constructed with the intention of making salient one or more patterns in a collection of data, where the patterns are associated with real-world phenomena about which the researcher is seeking evidence (Leonelli 2019).

While this constructive aspect of data practice is particularly salient in the context of data modeling, it is important to keep in mind the earlier point that even “raw” data are “made” (Leonelli 2016) through a process of inquiry, which itself is often carefully and deliberately designed (see also Tal 2012). There is a tendency to forget that data are, at least in this sense, *constructed*; instead, as noted in Section 2, data often are treated as unmediated windows on the world and consequently are granted some automatic epistemic privilege.^10^ The PR view embraces the idea that all data are constructed through a process of inquiry; however, we reject a more radical constructive thesis that would claim data are purely made-in-the-mind or have their contents freely chosen by scientists.^11^ We simply want to keep in view what should be uncontroversial: that data are the product of an *interaction* between a measuring device (or observer) and the world, and that both these, along with background conditions and the means by which data are recorded, can influence the content and character of the data produced. Data collection procedures are often carefully designed to manage these interactions so that sought-after information is obtained, but they are not always successful.

Thus, in contrast to the unmediated-window or mirror view of data, the PR view allows that data and data models can—and indeed often do—*misrepresent* aspects of the world to some extent. That is, they represent aspects of the world as being somewhat different from how they actually are. The recorded rain gauge reading of “35”, for example, might be a significant *under*estimate of the actual rainfall, due to wind loss; a wind-corrected value might be a slight *over*estimate of the actual rainfall, due to idealized assumptions of the correction procedure. Of course, even when data do misrepresent the world in salient ways, they might still be used coherently and successfully to answer particular questions of interest. Continuing with the rainfall example, the meteorologist who knows that her uncorrected rain gauge reading (representing a rainfall depth of 35 mm) is an underestimate of the true rainfall can nevertheless use that datum to successfully answer the question of whether more than 10 mm of rain fell. Likewise, if she knows that her wind-corrected data model (representing a rainfall depth of 41 mm) is an overestimate of the rainfall, she can still use that data model to successfully answer the question of whether more than 100 mm of rain fell.

As this example illustrates, a scientist who selects and uses a data model in her research does not have to accept that data model as *her representation* in the way suggested by van Fraassen (2008; see Section 2 above). That is, she does not have to accept the data model as a representation that, for her, is pragmatically indistinguishable from the world. Indeed, it is a mark of a good scientist that she explicitly acknowledges—and even tries to quantify—the limited accuracy, resolution and precision of her data. In the case of accuracy, this is often done with error-bars around the data points on a graph or, when data are presented in numerical format, by reporting them ± some amount, indicating the associated uncertainty. Van Fraassen (2008) recognizes that data models often incorporate such uncertainty information. But the problem remains: even a scientist who uses a data model that incorporates uncertainty information need not treat that data model as pragmatically indistinguishable from the world, since uncertainty estimates themselves are often recognized to be imperfect too—due to idealizations and simplifications employed in the uncertainty estimation procedure, or because some sources of uncertainty have not been taken into account yet, etc.

### Data adequacy-for-purpose

It remains to articulate the pragmatic dimension of the PR view. A number of philosophers have argued that the evaluation of theoretical models should consider not how close those models come to ‘mirroring’ real-world target systems but rather whether they represent their targets with *sufficient* accuracy in the respects that are *relevant*, given the *purpose* at hand (e.g., Teller 2001; Parker 2010). What matters, on this view, is that a model is *adequate for the purpose of interest*. Recent work in this vein has emphasized that it is not only how theoretical models represent their targets that can determine whether they are adequate for purposes of interest, but also other features of such models, such as their adaptability, their intelligibility, how computationally-demanding they are, and so on (Elliott and McKaughan 2014; Parker 2020a).

We propose that the same “adequacy-for-purpose” perspective be adopted when evaluating data and data models.^12^ On this way of thinking, the quality of some data or data model is relative to one or more purposes of interest; the question is not whether data are “good” or “bad”, where this is simply a matter of how close they come to perfectly mirroring reality, but rather whether they can be used to achieve the particular epistemic or practical aims that interest their users. The aims we have in mind are typically rather specific and circumscribed: determining how much rain fell last week in a given locale; testing a hypothesis about which of two species emerged first; uncovering patterns of bias in hiring practices at a particular set of companies; and so on.^13^*Data evaluation*, on this view, is an activity that seeks to determine whether a given dataset or data model is adequate for specified purposes, or to better understand the range of purposes for which it is adequate. While this view of data evaluation has been advocated in some scientific contexts, it has only begun to be examined by philosophers. For example, Bokulich (2018) in defending such a view, quotes paleobiologists who have explicitly recommended an adequacy-for-purpose approach when it comes to evaluating fossil data: “palaeontologists, like other scientists, should accept that their data are patchy and incomplete, and use appropriate methods to deal with this issue in each analysis. All that matters is whether the data are *adequate* for a designated study or not.” (Benton et al. 2011, emphasis in original).^14^ We urge that this perspective be adopted much more widely.

A basic question that such a view must address, however, is what it means for data to be adequate for a purpose. As Parker (2020a) notes, there are different senses in which a tool or resource can be adequate for a purpose. Here we present just two varieties of adequacy-for-purpose that we believe are often of interest: adequate-in-an-instance (adequacy_I_) and adequate-given-resources (adequacy_R_). The first, adapted from Parker’s discussion, is concerned with a particular envisioned or actual use of data: a dataset or data model *D* is *adequate*_*I*_*-for-P* just in case the use of *D* in instance *I* would (or would be very likely to) result in the achievement of *P*. Note that any instance of use of a dataset or data model will involve one or more *users U* and some way *W* of using the data, i.e., a *methodology*. To illustrate, suppose the purpose of interest is *P*_*1*_: estimating annual rainfall in a locale to within 10%. Though rain gauges are imperfect collectors of rain, if a meteorologist (*U*) simply adds together (*W*) the weekly rain gauge records (*D*) at her disposal, she might nevertheless obtain an annual rainfall estimate that is accurate to within 8% in that instance. That accuracy, since it is within 10%, is sufficient for her purpose and she would achieve her aim in that instance (the dataset *D* would be adequate_I_ for *P*_*1*_). But if her purpose had been *P*_*2*_: estimating annual rainfall to within 5%, then that dataset D would *not* have been adequate_I_ for that purpose. Whether the dataset is an adequate representation of rainfall at that location is not just an intrinsic property of the dataset, but rather depends on how it will be used and for what purpose.

A second variety of adequacy relates to the *possibility* of using data successfully, given a set of accessible resources: a dataset or data model *D* is *adequate*_*R*_*-for-P* just in case its user *U* has access to informational, technological, cognitive and practical resources *R*, such that there is some coherent way *W* that *U* could use *D* to achieve purpose *P*. The aforementioned rain gauge records might be adequate_R_-for-*P*_*2*_ if the meteorologist (*U*) has access to sufficiently-accurate data on wind speed over the year and a sufficiently-reliable equation relating gauge loss to wind speed (*R*), which she could apply to correct the gauge data for wind loss (*W*). That is, it is possible, given the resources available to her, for her to use the rain gauge data in a coherent way to successfully achieve the more demanding level of accuracy required by *P*_*2*_. Thus, data that are inadequate_I_ for a given purpose might still be adequate_R_ for that purpose.

The closely-related concept of fitness-for-purpose can also be employed in data evaluation, when the purpose of interest is one that can be achieved to a greater or lesser extent, such as *P*_*3*_: accurately estimating annual rainfall in this locale over the previous month. Again following Parker (2020a), we can think of such purposes as consisting of a rank-ordered set of achievements, *P* = {*P*_*min*_*,…,P*_*max*_}, where *P*_*min*_ corresponds to achieving *P* to an extent that the evaluator considers minimally-acceptable and *P*_*max*_ corresponds to achieving *P* to the maximally-desired extent (e.g. a perfectly accurate rainfall estimate, in the example given here). The *fitness*_*x*_*-for-purpose* of some dataset or data model *D* for purpose *P* is higher to the extent that *D* is adequate_x_ for higher-ranking members of the set *P*, where *x* picks out some variety of adequacy (e.g. adequacy_I_, adequacy_R_, etc.).^15^

Note that whether some dataset or data model is adequate-for-purpose in the senses articulated above depends not just on how it represents the world (i.e., a representational target), but on whether it stands in a suitable relationship with that representational target, a data user, a methodology (or set of available methodologies/resources), and background circumstances *jointly.* These can be thought of as dimensions of a *problem space,* in which the goal is to achieve the purpose *P* of interest (Parker ibid.).^16^ The different dimensions constrain, and in some cases determine, what properties data and data models need to have if they are to be adequate-for-purpose. These properties include—but are not limited to—the accuracy (and precision and resolution) with which data and data models represent some aspect of the world. Depending on the purpose, they can also include, for example, whether data are easily portable, whether they are accompanied by particular metadata, whether their format makes relevant patterns salient to users with particular cognitive abilities and background knowledge, etc. The latter, for instance, might be very important if a dataset or data model is to be inspected by users who seek to develop explanations of phenomena; users need to be able to ‘see’ the relevant explanatory information if they are to succeed in achieving their purpose.

### Illustrating the PR view

The simple rain gauge example above is useful for introducing the PR view. However, since most uses of data in science are not so simple, in this section we illustrate key elements of the PR view with a more complex example from scientific practice.

Shaun Marcott et al. (2013) developed the first reconstruction of the evolution of global temperature over the Holocene period, from 11,300 years ago to the present. They started from published temperature reconstructions for 73 sites around the globe, produced from a variety of proxy indicators, including marine and terrestrial fossils, isotopes in lake and ocean sediments, ice cores, etc. These temperature records, each of which spanned most or all of the Holocene, were in some cases recalibrated by Marcott et al. to reflect updated methods for converting radiocarbon dates to calendar dates.^17^ A Monte-Carlo methodology was then used to generate 1000 realizations of each record, linearly interpolated to constant time spacing; each realization constituted a possible evolution of temperature at the site, given uncertainties associated with dating the proxy indicators and inferring temperatures from them. These were combined to produce 1000 reconstructions of global temperature evolution over the Holocene. Calculating the mean and standard deviation for these 1000 global reconstructions produced a best-estimate reconstruction and an estimate of its associated uncertainty, respectively. From this, Marcott et al. also estimated the statistical distribution of global temperature during the Holocene period. These steps were repeated with different methodological choices (e.g., different ways of combining the local reconstructions to arrive at a global one) to test the sensitivity of the results.^18^

From the perspective of the PR view, we can see several layers of *representation* in the Marcott et al. study. The key outputs of the study were two types of data products that represented (a) the evolution of global temperature over the course of the Holocene and (b) the statistical distribution of global temperature during that period, inferred from (a). As explained above, these were constructed in a complex way from a set of already-available data products, each representing the evolution of temperature in a particular locale. Each of these data products, in turn, was ultimately developed (perhaps with several additional layers of data modeling in between) from data representing particular features of fossils or sediments or ice cores, etc.; the transformation of these data about fossils into data about temperature was a crucial first step.

Considerations of *adequacy-for-purpose* figured prominently in both the published paper and subsequent discussion of it in the blogosphere. A key aim of the Marcott et al. study was (*P*) to determine how unusual recent global temperatures are, relative to the rest of the Holocene. As Marcott et al. say: “Because the relatively *low resolution* and *time uncertainty* of our data sets should generally suppress higher-frequency temperature variability, an important question is whether the Holocene stack *adequately* represents centennial- or millennial-scale variability” (p. 1198, emphases added). The concern was that, if high-frequency variability was smoothed over too much, then the analysis might substantially overestimate the extent to which recent global temperatures are unusual, relative to the rest of the Holocene. To probe this, Marcott et al. applied their reconstruction methodology to synthetic data containing high-frequency variability, allowing them to estimate the extent to which such variability would be missed.^19^ This led to a revised estimate of the statistical distribution of temperature over the Holocene period, which took account of this estimated missing variability. Using this revised data product, they concluded that recent temperatures were warmer than during ~72% of the Holocene, rather than during ~82% of it, as implied by their standard reconstruction. Here we see researchers focusing their evaluative and corrective efforts on particular aspects of their data modeling procedure that might render their data products inadequate for the purpose at hand (P); with a different purpose of interest, attention might well have been focused on other aspects of the data and data products.

The Marcott et al. study thus exemplifies three important elements of the PR view: the *representational* character of data and data models; the fact that they are not ‘given’ but rather *constructed* through a process of inquiry that in many cases is quite complex; and a focus, when evaluating data and data models, on their *adequacy for particular purposes*.

## Three implications for data evaluation

Having introduced the basic elements of the pragmatic-representational (PR) view of data, we next want to highlight three important implications for data assessment in practice, related to misrepresentation (Section 4.1), context-sensitivity (Section 4.2) and complementary use (Section 4.3).

### Misrepresentation

A clear implication of the PR view is that, when evaluating data, the fact that they misrepresent aspects of the world in various ways should not automatically “count against” them; misrepresentation is problematic only if it renders data inadequate for the researcher’s purposes. Philosophers advocating a pragmatic, representational perspective on theoretical modeling have emphasized the same point in that context (e.g., van Fraassen 2008; Bokulich 2016; Parker 2020a). Just as misrepresentation need not render a theoretical model inadequate for a purpose of interest, it need not render data inadequate either; having data that represent the world in a highly-accurate way is not always necessary. This point is more familiar today than ever, with the rise of “big data” and machine learning methods. Such methods often succeed in extracting relationships that are useful for predictive purposes, even when the data stream under analysis is noisy, error-ridden, etc. Here, the sheer volume of the data allows the algorithm to learn some useful predictive relationships, despite far-from-perfect data.

Examples can readily be found in more traditional scientific contexts as well. Suppose a scientist wants to (*P*) test the hypothesis that the non-avian dinosaurs went extinct due to an asteroid impact. She plans to do so by seeing whether the two events are temporally coincident according to radiometric dating of samples associated with the two events. Testing for the coincidence of the two events does not require that estimates of their absolute ages (i.e., measured in calendar years) be highly accurate. Data from a single high-*precision* dating method that can be applied to both the extinction event and the impact event could suffice; systematic biases in that dating method (such as an incorrect value for the relevant decay constant) could skew the absolute ages of the events, making them off by several million years, but as long as both ages reflect the *same* systematic offset, the question of whether they are temporally coincident could still be successfully answered (for a discussion of precision and accuracy in radiometric methods see Bokulich 2020a). The radiometric data would be adequate for the researcher’s purpose, despite their significant inaccuracy.^20^

In fact, there can be reasons to choose a less-accurate and/or lower-resolution dataset over a more-accurate, higher-resolution one. Sometimes the reasons are pragmatic: an answer to a question is needed in a particular time frame, and a good-enough answer can be obtained more quickly from the less-accurate or lower-resolution data (see Elliott and McKaughan 2014 for a similar point). Indeed, the more-accurate, higher-resolution data may be inadequate for the researcher’s purpose, insofar as it is infeasible for the researcher to analyze or process the data on the timescale required. In other cases, reasons for choosing a less-accurate, lower-resolution dataset can stem from the cognitive capacities or limited background knowledge of the data users. Such a preference is especially plausible, for instance, when it comes to using data for pedagogical or explanatory purposes. This is analogous to the way in which simpler theoretical models can be preferable when the aim is explanation and understanding of the behavior of complex systems and phenomena, because the simple models’ behavior is easier for researchers to explain and understand (e.g., Bokulich 2008; Kuorikoski and Ylikoski 2015).

Of course, this is not to deny the general value of having data whose accuracy, precision and resolution are high. Such “high-fidelity” data can be expected to be adequate for a broad range of purposes. The point is simply that higher-fidelity data are not always preferable; lower-fidelity data can sometimes have greater fitness-for-purpose.

### Evaluation in context

A second significant implication of the PR view is that data cannot be evaluated independently of their context of use. The point here is not just that evaluations of data quality can vary with the purpose of interest; as we emphasized in Section 3.2, the properties that data need to have if they are to be adequate depend on other dimensions of the problem space as well, notably the methodology that the data user will employ (or the set of methodologies available to her) and, in some cases, the user’s cognitive and other abilities. An analogous observation is made by Parker (2020a), when advocating an adequacy-for-purpose view in the context of theoretical model evaluation; she argues that, while some philosophers have suggested that model quality is purpose-relative (e.g. Teller 2001; Giere 2004), under an adequacy-for-purpose view it is relative to a broader *problem space*.

Among other things, this context-relativity of data quality means that often one cannot “read off” from a purpose alone a set of properties that the data or data models must have if they are to be “good enough”. For example, if our aim is to order days of a month from most to least rainfall, it does not follow that we need data that, when taken at face value, produce the correct ordering; the ordering implied by the data might be quite inaccurate, due to some rainy days being very windy and others not, yet those data could still be adequate for our purpose if our methodological toolbox includes a means of correcting for wind loss. This point is closely related to Tal’s (2012) challenge to van Fraassen’s (2008) “criterion for the physical correlate of measurement.” Tal illustrates how the same state of a measuring apparatus can be mapped to different measurement outcomes, depending on assumed background conditions, known interfering factors, and so on. Depending on the mapping procedure (i.e., the methodology) to be employed—part of the *context of use*—the properties that data and data models will need to have to be “good enough” for a given purpose might well vary. Similarly, it can depend on other dimensions of the problem space, including the data user and the background circumstances in which the data will be used.

### Complementary uses

A third important implication of the PR view is that data and data models that are understood to represent the same aspects of the world need not be seen as competitors. Once again, this parallels the situation in theoretical modeling, where different models of the same target system need not be in competition with one another; they might be useful for different purposes or might be complementary in various other ways (e.g., Parker 2006; Bokulich 2013). Here we discuss two ways in which datasets representing the same aspects of the world also can be complementary.

First, like theoretical models, datasets that represent the same aspects of the world can be suited to different purposes. Consider two national rainfall datasets that have different spatial resolution; the high-resolution data might be required for quantifying changes in rainfall in particular cities, while the somewhat lower-resolution data might be preferable for discerning broad patterns of change at regional scales (e.g., due to the data’s being easier to work with or avoiding unnecessary detail, etc.). Ideally, these datasets would be consistent with one another, in the sense that regional-scale rainfall that is inferred using the high-resolution dataset would be within the uncertainty bounds associated with the estimate inferred from the lower-resolution dataset, but even this is not strictly necessary for the datasets to be complementary resources, where each is better than the other for some purposes.

Second, datasets and data models that represent the same aspects of the world can be complementary insofar as they *jointly* serve a particular purpose. For example, a set of data products, each representing the evolution of paleoclimatic temperatures but developed from different types of proxy indicators (e.g., tree rings vs. ice cores), can provide valuable insight into the extent of current uncertainty about those past temperatures. The same is true of sets of data products representing the evolution of twentieth century global temperatures, which are produced from the same thermometer data, but with different methodologies for processing those data: “Multiple [data] products are the only conceivable way to get even a simple estimate of the structural (methodological choices) uncertainty; we need to attack the problem from many different a priori assumptions to create an ensemble of estimates” (Thorne et al. 2011, p. ES44). In cases like these, the alternative data models are used together for a single purpose, namely, uncertainty exploration or quantification. Consequently, their evaluation should focus on how well they *together* serve this purpose.

## The dynamics of data: reusing and repurposing

Our discussion so far has centered on the use and evaluation of data at a given point in time. Importantly, however, neither the assessment of data adequacy nor the choice of purpose need be static. Efforts can be made to help data better serve a particular purpose, and the range of purposes for which data are adequate can evolve over time as new knowledge and techniques become available. Here we describe these “dynamics of data” in terms of the concepts of data reuse and repurposing.^21^ By *data reuse* (Section 5.1) we mean using data again for essentially the *same* purpose for which they were used previously. Reuse involves a reexamination, reanalysis, or reprocessing of a data set with the aim of better answering a question already addressed by those data. By contrast, *data repurposing* (Section 5.2) is using pre-existing data for a *different* purpose than was initially envisioned by the data collectors or primary data users.

There are various reasons why scientists might reuse or repurpose data. Reuse sometimes stems from an interest in whether the results of a study can be replicated or reproduced. Even if no errors per se were made, new (or different) data modelling techniques might yield better results for the purpose of interest (e.g., might allow for a clearer signal to be extracted from noisy data, even if the basic conclusion of the study is unchallenged). Moreover, some data sources are ephemeral; a particular hurricane, for example, lasts only so long, after which there is no possibility of re-observing it. In the case of repurposing, the impetus is often simply the recognition that additional, interesting scientific questions can fruitfully be addressed using existing data. More generally, considerations of efficiency often play a role: data collection can be very expensive, difficult, and time-consuming, making the reuse or repurposing existing data an attractive option.

These reasons help to explain the tremendous push of many scientific, grant, and government agencies towards open data principles, which require that scientists make their data freely available online in community databases, such as the Paleobiology Database, the Cancer Imaging Archive, HEPData, or NASA’s EarthData. These community databases allow for multiple reuses and repurposings of the data, as well as the integration of many different data sources by users. Similarly, the growing movement to rescue so-called “legacy data” or “dark data” only makes sense in light of the dynamic reusing and repurposing of data. Legacy data are those whose method of collection or storage inhibits their continued use. To make them usable—either for reuse or repurpose—requires that the data be *re-curated*, which can itself involve many steps, such as changing the substrate of the data (e.g., from analog to digital), re-standardization, or semantic reinterpretation. These movements (open data, community databases, data rescue) are increasingly facilitating data reuse and repurposing across a range of fields. An important implication of this, we argue, is that data sets increasingly have a kind of “evolutionary” history that can be highly relevant to their evaluation and use (Section 5.3).

### Data reuse

As defined above, data reuse involves the reexamination, reanalysis, or reprocessing of a data set, so that it can be used for essentially the same purpose for which it had previously been used. The aim is usually to arrive at improved data models—ones that are more likely to be adequate for the purpose(s) of interest (or that have greater fitness-for-purpose). Reuse of data occurs because science itself is a process—a fallible enterprise that often increases its epistemic reliability through iteration; this applies no less to data than it does to theory. Sometimes, reuse is prompted by a change in background theory or standards. For example, Bokulich (2020a) discusses how radiometric data need to be periodically reprocessed, as measurement standards and the empirically determined values of decay constants change. Other times, researchers come to recognize that interfering factors were not sufficiently controlled for, or adequately corrected for, previously.^22^ In still other cases, new or alternative statistical data processing methods become available, which have advantages over those previously used. Finally, there can be other sorts of data enhancements that facilitate the reuse of data for the purpose of interest, such as new methods of data interpolation or the integration of the data set with other data sets.

In fact, in many scientific fields there is an *iterative process aimed at data model improvement*. Here we present just one example, involving stellar radial velocity (RV) data used to detect exoplanets*.*^23^ RV data can record changes in the radial component of the velocity of a star due to the gravitational pull of an unseen exoplanet; the starlight is blue-shifted when the star is pulled towards us by the planet and red-shifted when it is pulled away. Initial analysis of RV data collected by the HARPS (High Accuracy Radial velocity Planet Searcher) spectrograph at the European Southern Observatory in Chile indicated three exoplanets orbiting the red dwarf star Gliese 581 (GJ 581) (Udry et al. 2007). HARPS data, which consisted of 119 velocities collected over 4 years, was reused by Vogt et al. (2010), who combined it with another RV dataset obtained from the HIRES spectrograph on the Keck I telescope in Hawaii; HIRES data consisted of 122 velocities obtained over 11 years. Their analysis of the combined data sets indicated not just three, but six planets orbiting GJ 581. They used the two data sets (HIRES and HARPS) not just collectively, but also to probe how many of those planets were independently confirmed by each data set, emphasizing that “inter-team comparisons on stars like [Gliese 581]. . . will be crucial to quantifying the true precision limits of any team’s data sets” (ibid). This illustrates the complementary use of multiple datasets about the same target, highlighted in Section 4.3.

Subsequently, Baluev (2013) reanalyzed the same HARPS and HIRES data and argued that they contained a significant correlated red-noise component, which had not been accounted for by Vogt et al. (2010). Since the source of correlation was unknown, the data processing path was bifurcated, producing two datasets reflecting different noise models. On Baluev’s analysis of the noise-corrected data, two of the exoplanets previously thought to orbit GJ 581 were deemed artefactual, and the existence of a third (GJ 581 *d*) became uncertain; instead of six exoplanets orbiting GJ 581, there were likely only three or four. More recently, building on Baluev’s red-noise corrected version of the HARPS and HIRES data sets, Robertson et al. (2014) investigated the period of stellar rotation for GJ 581 and showed that, when the data are corrected for this stellar activity, the exoplanet *d* that was hanging in the balance effectively disappears, leaving just three planets. These efforts, which involve repeatedly reusing the HARPS and HIRES data sets to answer the *same* question of how many exoplanets are orbiting GJ 581, are still ongoing. Given questions about these methods, Vanderburg et al. (2016) are using computer simulations and synthetic data to explore the reliability of various data correction methods that disentangle the RV signal of the exoplanets from the signals caused by stellar activity (e.g., due to starspots and stellar rotation), learning more as they go.

This sort of iterative development of datasets and data models, where the same data are re-processed and re-analyzed so that they can better serve a particular purpose of interest, can be seen in many other scientific contexts too. In some cases, it is reflected in the very labels given to datasets, marking them as particular *versions*, as is also commonly done for theoretical models (e.g. computer simulation models). In climate science, for example, global temperature datasets are often labeled in this way; successive versions the HadCRUT dataset, developed over multiple decades and reflecting various methodological innovations, are HadCRUT1, HadCRUT2, HadCRUT3, etc. (see Osborn and Jones 2014). The ever-evolving state of scientific, technological, and statistical knowledge means that, even with the same data (e.g. HARPS/HIRES) and the same questions being asked of them (How many exoplanets are orbiting GJ581?) there can be differences in the conclusions drawn. The hope is that, through iterative reuse, researchers develop data models that better serve the particular purposes that interest them—such as determining the number of exoplanets around a star or quantifying the warming of Earth’s surface temperature during the twentieth century.

### Data repurposing

While data *reuse* involves using the same data to answer the *same* question, data *repurposing* involves using the same data to answer a *different* question. This repurposing can take several forms. First, and most straightforwardly, a data set about some quantity, *x*, can be recognized as adequate for (or at least relevant to) additional *x*-related purposes, though perhaps requiring some further data wrangling.^24^ Second, it might turn out, as in the case of derived measurement or data conversion (Bokulich 2020b), that data about quantity *x* can be converted through well-established laws or relations into estimates of another quantity *y.* For example, data recording the travel time of light or an acoustic signal (*t*) can be converted into data about distances (*km*). Third, even if there is not a direct or exact conversion from quantity *x* to another quantity *y*, it may be that *x* can be taken as a rough proxy for *y*. An example is the use of leaf shapes recorded in fossils as a proxy for paleoclimate temperatures, with smoother leaf edges indicating warmer climates and more jagged “toothed” leaf edges indicative of cooler climates (e.g., Royer et al. 2005). In these ways, data that were collected with certain purposes in mind can be repurposed to serve others.

A striking example of this is a recent repurposing of data gathered from the Mars rover Curiosity. Curiosity was equipped with a set of three-axis gyroscopes and accelerometers for measuring changes in velocity and orientation—data which together made up the rover’s Inertial Measurement Units (RIMU) system for navigation. These data were essential for allowing the rover to remain right-side-up and balanced as it moved through the steep and rocky Mars terrain. Curiosity collected these RIMU data and beamed them back to Earth as scientists directed the Rover to cross the Gale crater and climb the foothills of Mount Sharp. Geoscientist Kevin Lewis et al. (2019) repurposed these data, initially used for navigation, in order to help resolve a scientific debate about the origin of Mount Sharp: Was the crater initially filled with sediment and then subsequently eroded away leaving behind the mountain, or was the crater never filled, and instead the mountain was constructed by wind deposition and other processes? Answering this question required gravimetric data—measurements of the gravitational field (*g*) at different places on the planet’s surface—which could be used to infer the structure and density of the planet’s subsurface. While Curiosity’s payload was not equipped with a gravimeter, Lewis et al. recalibrated and reprocessed the RIMU data, applying corrections for purpose-relevant confounding factors, in order to obtain estimates of gravitational changes with elevation. From these data, Lewis et al. inferred that the underlying rock was of low density (high porosity), indicating that the crater could not have been buried up to the height of Mount Sharp (5 km), which would have resulted in significantly more compactification, and hence higher density.

Thus, data initially collected for the purpose of navigation were repurposed for a completely different end, that is, for measuring gravitational changes with elevation in order to determine the density of subsurface rock. This illustrates vividly a point made earlier in Section 3.1: oftentimes datasets can—if appropriately processed—provide evidence regarding a variety of different claims, if the knowledge and other resources needed to extract the relevant information is available to the scientist; the evidential value of the data is constrained, but certainly not fixed. From the perspective of the PR view, examples like these illustrate that data can be adequate for a wide range of purposes beyond those for which they were originally collected.

### A phylogeny of data: trees, not hierarchies

Insofar as data are frequently reused and repurposed, we can expect that datasets and data models will often have a kind of evolutionary history or phylogeny. Their current incarnations will be a product of a prior sequence of modifications, as data are produced, processed, and reprocessed in different ways. Some of these modifications will be cumulative, while others will bifurcate a data lineage, resulting in datasets being developed along different paths as needed to adapt the data for (increase their fitness for) different purposes. Choices made at one stage—such as to smooth data in a particular way or exclude particular outliers—will sometimes become “generatively entrenched” (Wimsatt 2007), shaping and constraining the future development of those data models.^25^ If one were to map the history of a dataset from its origin as “raw” data at the time of collection, up through its various wranglings, corrections, and other modifications to its different uses as evidence, the resulting picture would in many cases be a complex, branching tree structure, reflecting the evolution of the dataset (or data model) as scientists learn how to best extract the information needed for particular purposes.

Note that the tree structure we are identifying here is different from Suppes’s (1962) hierarchy of models, discussed briefly in Section 2. Suppes’s hierarchy was concerned with the synchronic problem of how a scientific theory relates to the world, or more precisely how Tarskian models of theory are related to Tarskian models of the data through a succession of intermediary models, such that the two can be compared. What we are instead calling attention to here is the diachronic history of the data models themselves—a process akin to descent with modification, as data are reused and repurposed by various researchers. Importantly, on this picture, data models are not simply “made more accurate” as they evolve; the respects in which they become more (or perhaps less!) accurate, and more generally the ways in which they are changed, are shaped by the particular epistemic and practical goals of the researchers involved.^26^ As we have illustrated, researchers can develop a dataset in different ways to serve different purposes. This can be seen with both the wind-influenced-rain-gauge dataset, presented in Section 3.1, and the Mars rover dataset repurposed for gravimetry data about subsurface density, discussed above. With different purposes of interest, the development of the dataset might focus on correcting for a *different* set of confounders, or might set a *different* threshold for discarding outlier data, and so on.

This in turn suggests that information about the historical lineage of a data set—including any original purpose for which it was collected and any modifications it has subsequently undergone—can aid its appropriate use in important ways. For instance, knowing that the original process of data collection was optimized to serve a particular purpose can provide insight into which sorts of errors the data collectors might have been especially careful to avoid, as well as which other sorts of errors the data are likely to contain.^27^ Zimmermann (2008), in her ethnographic study of ecologists who repurpose data that they did not collect themselves, found that these scientists were well aware of the value of such information. She writes, “Ecologists discussed the importance of knowing that the purpose for which data were gathered guides appropriate reuse^28^ of them. . . . Research purpose dictates methodological choices, which in turn affects the data that are generated” (Zimmermann 2008, p. 642–3). The purpose for which data are being collected can shape the scientist’s choice of which measuring instrument and methodology to use in collecting that data, and thereby influence the qualities of the data produced. Hence, in cases where data are collected with a particular purpose in mind, this can be valuable information to include in the accompanying metadata (i.e., data about data).^29^ Clearly, awareness of any modifications that have been made since the data were originally collected can also be relevant to determining whether the dataset, at its present state of development, is adequate for a given purpose.^30^ Indeed, a phylogeny of the data can be invaluable not only for evaluating whether a data model, taken at face value, is adequate for a purpose of interest, but also for understanding what further data wrangling or enhancement might be required to develop a data model that has *greater* fitness for the purpose that interests us.

## Concluding remarks

We have defended a novel *pragmatic-representational* (PR) view of data and data models, which avoids the problematic assumptions of both the naive mirroring view and the Suppesian set-theoretic view. Unlike these commonly-assumed views, the PR perspective leaves room for the complex iterative interplay between researchers and the world in producing and developing data and data models. The PR view understands data and data models to be representations of various aspects of the world. Minimally, they are *taken to be about* processes thought to be involved in their production and, in many cases, they have more specific representational content. Recognizing data as representational, however, does not commit one to the view that their evidential value is fixed. As we have illustrated, the same data can be informative about various aspects of the world, though *which* aspects are of course constrained by the processes involved in the data’s production.

While an adequate epistemology of data must leave room for data to *misrepresent*, the pragmatic element of our PR view emphasizes that misrepresentation is not necessarily problematic. The central insight of the PR view is that data and data models, like theoretical models, should be evaluated in terms of their *adequacy or fitness for particular purposes,* rather than relative to some ideal standard of perfect representation. Moreover, whether data are adequate-for-purpose depends not just on how they represent aspects of the world, but also on how they relate to other dimensions of a broader problem space, such as the data user’s abilities, resources, and the methodology to be employed.

The PR view of data is also a *dynamic* view: neither the assessment of adequacy nor the choice of purpose need be fixed. As we illustrated, a dataset that is inadequate for a given purpose when one set of resources is available or employed, can become adequate for that purpose with access to additional resources that allow for further data processing, such as data filtering or data integration. Researchers often work hard to build improved data models—ones that better serve their purposes—from a given set of “raw” data. In addition to this data *reuse*, data can be *repurposed*: data that were initially collected for one purpose can be retooled, through processes such as data conversion, to serve a range of additional purposes. The upshot of this reusing and repurposing is that datasets often have a kind of evolutionary history (or phylogeny), which can be highly relevant to evaluating their adequacy or fitness for purposes of interest. Information about the original purpose for which the data were collected, as well as key stages in their lineage (e.g., filtering, processing, etc.), can be valuable for future users of the data. This underscores that such information should be included in a dataset’s associated metadata.

In addition to advancing the philosophy of data, the PR view may also be of use to practicing scientists. First, the PR view urges that scientists think of data not as detached and self-sufficient elements of reality, but rather as records of a process of inquiry; hence, their origin and history become relevant to their assessment and use in practice. Second, although some scientists take the view that the evaluation of data should be tied to purpose, many seem to implicitly subscribe to something like the mirroring view, according to which data are evaluated merely as accurate or inaccurate, good or bad, *tout court*. A more fruitful assessment would take into account the particular planned use of the data, and instead ask how various features of the data—not just their accuracy, resolution and precision, but also their format, available metadata, previous processing, etc.—bear on their adequacy or fitness for that particular purpose. An explicit recognition of the PR view could thus help avoid debates where scientists are speaking past each other in their assessments of data, because they have different purposes in mind. Finally, by recognizing the potential for data models to be refined over time—both to better serve existing purposes (reuse) and to be used for purposes beyond those for which they were originally collected (repurposing)—our view reinforces current movements calling for open data and data rescue.

We see numerous opportunities for further research. One project involves further integrating the PR view of data with recent work in the philosophy of measurement (metrology), especially the model-based view of measurement developed by Tal (2012). Other promising avenues include the following: performing detailed case studies of the ways in which adequacy considerations shape choices in data model development; tracing the phylogenic histories of important datasets and how their evolution was shaped by researchers’ purposes and by other dimensions of the associated problem space; relating these evolutionary histories to emerging discussions of “data journeys” and how data “travel” (Leonelli 2016; Leonelli and Tempini 2020); exploring the extent to which distinctive challenges arise when evaluating the adequacy of data of different types (e.g., quantitative versus qualitative), in different fields (e.g., physics versus sociology), and with respect to different types of purpose (e.g., understanding versus prediction); and analyzing particular scientific disputes over data through the lens of the PR view. Through such investigations, our philosophical understanding of data and data models can begin to catch up with our understanding of theoretical models.

## Footnotes

1 Though for a preliminary step in this direction see Bokulich (2018).

2 We understand data models to differ from what are sometimes called “data-driven” or “phenomenological” models. The latter, while developed in light of data, typically take the form of mathematical equations that are meant to characterize somewhat general relationships among aspects of the target. Data models, by contrast, typically take the form of datasets, graphs or images and pertain to what happened at particular times and places.

3 The notion of abstraction here in the context of data is similar to the notion of abstraction in theoretical modeling; one is replacing the thing in the world with a representation, and that process will always involve some omission.

4 For a discussion of various conceptions of scientific theories (e.g., syntactic, semantic, and pragmatic) and critiques of the semantic conception see, for example, Winther (2016) and references therein.

5 In her more recent work Leonelli (2019) indicates that her views on Suppes’ notion of a data model have begun to shift (p. 25, fn 31).

6 As should be clear, in this paper we are *rejecting* the semantic view of theories and concomitant Tarskian view of models, as it is an inadequate account of both theoretical models and data models for the sorts of scientific cases we are interested in.

7 This view is narrower than that of Leonelli (2016), insofar as Leonelli counts as data any material artefact that is taken to be potential evidence for one or more claims about phenomena. Thus, for example, she allows that a mice colony could constitute data, if it is taken as potential evidence for claims about a link between genes and behaviour. We prefer to maintain a distinction between a system or phenomenon that is under study (e.g. the mice colony) and data about those phenomena or systems (e.g., records of the observed behavior of the mice). Woodward (2010) allows that both the results of a process of inquiry (e.g., an instrument state) and a record of those results (which can itself be considered a result) can be data; this is broader than our characterization but narrower than Leonelli’s. We do not think a tremendous amount hangs on which of these characterizations one chooses. The choice does not, for instance, prevent our reaching a number of other conclusions about data and data models that align with those of Leonelli, Woodward and others (see citations throughout our discussion).

8 Precision thus is a property of the process of inquiry, while accuracy is a property of data themselves.

9 Leonelli (2019) grants that data models can be representations, but denies that data themselves are (in general) representations; hence she takes a slightly different view than we do here, as noted above.

10 For an analysis of the epistemology of theoretical models with an emphasis on how models are constructed, see Boon (2020). The constructed nature of theoretical models (as opposed to data and data models) seems less often forgotten, though even in the case of theoretical models there is sometimes talk of the ‘fallacy of misplaced concreteness’ (Whitehead 1925), in which abstract objects like models are treated as, or mistaken for, reality.

11 Data collected by introspection alone may be a special case; we will not worry about it here.

12 To be clear, we are not making an argument based on an *analogy* with theoretical models; our claim is that features of data and data models themselves speak in favor of adopting the adequacy-for-purpose view.

13 This can be contrasted with generic aims or purposes, such as “predicting” or “explaining” or “discovering new phenomena” or “making good decisions”. A further issue is whether some epistemic or practical aims should be considered illicit or out-of-bounds, e.g. if the aim is to use data to mislead people or coerce or manipulate them. We do not have space to address this here; we assume in the present discussion that the purposes of interest are standard scientific ones, consistent with the scientific values of knowledge production, understanding, truth, etc.

14 See also Zumwald et al. (2020) in the context of climate science.

15 Given that fitness-for-purpose is analyzed in terms of adequacy-for-purpose, most of the remainder of our discussion will be framed just in terms of adequacy-for-purpose, for simplicity.

16 As hinted above, the notions of adequacy-for-purpose offered here can easily be adapted for evaluation of methodologies and perhaps other entities/resources too. That is, we could ask whether a *methodology* is adequate-for-purpose, given a data user, a dataset and the circumstances in which the methodology will be deployed. This seems close to what Mayo (2018, p.297) has in mind when she speaks of the “adequacy for a problem” of statistical modeling assumptions and techniques in the context of data modeling.

17 For a philosophical discussion of the recalibration of radiocarbon dates see Chapman and Wylie (2016) or Bokulich (2020a).

18 This is a simplified account of the methodology; a more detailed description can be found in the published paper and in a subsequent FAQ post on the blog RealClimate. See http://www.realclimate.org/index.php/archives/2013/03/response-by-marcott-et-al/

19 For a discussion of how synthetic data can be used to test the adequacy of data correction methods, see Bokulich (2018) and Parker (2020b).

20 If instead the researcher used data from two *different* radiometric methods (e.g., an argon-argon date and a uranium-lead date) for the two different events, and those dating methods had *different* systematic biases, then the data would not be adequate for establishing the coincidence of the two events.

21 Although the terms “data reuse” and “data repurpose” are sprinkled throughout the data science literature, there does not appear to be any firm consensus in how either of these terms is used, including whether they are synonymous (e.g., compare Zimmermann 2008, Berman 2015, Woodall and Wainman 2015, and van de Sandt et al. 2019). Here we take them to be conceptually distinct, as we explain.

22 As Tal (2012) argues, successful measurement requires a model of the measuring process; as scientists learn more about their instruments, the world, and the measurement interactions between them that produce the data, their model of the measuring process can be refined. This can lead to refined or changed understanding of data.

23 This example is briefly discussed by Berman (2015), p. 67.

24 *Data wrangling* is a general term for activities such as cleaning, structuring, or otherwise optimizing a data set for use.

25 See Lenhard and Winsberg (2010) for a similar point about choices made in the development of complex computer simulation models (i.e., a kind of theoretical model).

26 This loosely—but only loosely—parallels the way in which the traits selected for in biological evolution depend on the environment of the population.

27 Here too, there are parallels with theoretical models; see e.g. Parker and Winsberg (2018).

28 Zimmerman uses the term “reuse” to mean what we here call “repurpose” (ibid., p. 634).

29 Even when researchers claim to have no specific purpose for their data in mind, they nonetheless must make choices about what instruments or methods to use in the collection of that data, which will influence the character of the dataset.

30 Such metadata are important for creating what Nora Boyd (2018) calls “enriched evidence”, by which she means “evidence enriched by auxiliary information about how those lines were generated . . . [including] metadata regarding the provenance of the data records and the processing workflow that transforms them” (2018, pp. 406–407).
